# Surgical System Efficiency and Operative Productivity in Public and Private Health Facilities in Ethiopia: A Cross-Sectional Evaluation

**DOI:** 10.9745/GHSP-D-22-00277

**Published:** 2024-02-28

**Authors:** Manuel Kassaye Sibhatu, Edlawit Mesfin Getachew, Dawit Yifru Bete, Senedu Bekele Gebreegziabher, Tsegaye Hailu Kumsa, Mulatu Birru Shagre, Kassa Haile Merga, Desalegn Bekele Taye, Hassen Mohammed Bashir, Mikiyas Teferri Yicheneku, Wuletaw Chanie Zewude, Akililu Alemu Ashuro, Tigistu Adamu Ashengo, Berhane Redae Meshesha

**Affiliations:** a Jhpiego, Addis Ababa, Ethiopia.; b Addis Ababa University, Addis Ababa, Ethiopia.; c Armauer Hansen Research Institute, Addis Ababa, Ethiopia.; d Ministry of Health of Ethiopia, Addis Ababa, Ethiopia.; e Saint Paul Hospital Millennium Medical College, Addis Ababa, Ethiopia.

## Abstract

**Background:**

Global and local health organizations track surgical system efficiency to improve surgical system performance using various efficiency metrics, such as operating room (OR) output, surgical incision start time (SIST), turnover time (TOT), cancellation rate among elective surgeries, and in-hospital surgery wait time. We evaluated the surgical system efficiency and factors affecting the efficiency in health facilities across Ethiopia.

**Methods:**

A cross-sectional study design with retrospective record review was used to evaluate the surgical system efficiency in 163 public and private health facilities in Ethiopia from December 2020 to June 2021. Experienced, trained surgical clinicians abstracted efficiency data from service registers and patient charts using a pretested tool. A bivariable and multivariable regression analysis was conducted.

**Results:**

In the study facilities, 84.11% of the operating tables were functional, and 68,596 major surgeries were performed. The aggregate OR output in both public and private health facilities was 2 surgeries per day per OR table. Operating productivity was shown to be affected by first-case SIST (*P*=.004). However, of the total 881 surgery incision times audited, 19.86% of the first-of-the-day elective surgeries started after 10:01 am. The SIST was strongly associated with an in-hospital wait time for surgery (*P*=.016). The elective surgery cancellation rate was 5.2%, and aggregate mean TOT was 50.25 minutes. The mean in-hospital surgery wait time was 45.40 hours, longer than the national cutoff for wait time. In a bivariable analysis, the independent variables that demonstrated association operating room productivity were then inputted into a multivariable regression analysis model. However, none of the predictor/independent variables showed significance in the multivariable regression analysis model.

**Conclusion:**

The volume of surgery and overall OR productivity in Ethiopia is low. This calls for concerted action to optimize OR efficiency and improve access to timely and safe surgical care in Ethiopia and other LMICs.

## Abstract (precis)

The surgical system in Ethiopia in both public and private health facilities was inefficient, requiring immediate action to improve timely access to safe surgical care.

## Teaser

**Key Findings:**

- In both public and private health facilities in Ethiopia, more than 84% of operating tables were functional. However, surgical system efficiency was low at an average productivity of 2 surgeries per operating room table per day.
- Predefined surgical incision start time, a strong predictor of operative productivity, was reported in only 31% of facilities. Of the surgery incision times audited, nearly 20% were delayed.
- First-case incision start times before 9:00 am had a significant association with shorter in-hospital surgery wait times.

**Key Implication:**

- Health organizations should monitor the productivity of surgical systems and introduce efficiency optimization tools to improve the timeliness and safety of surgical care.

## INTRODUCTION

To meet universal health coverage targets, countries need to invest more to enhance health care quality dimensions and accelerate access to safe, efficient, and affordable surgical care to reduce the disproportionately high mortality and morbidity rates associated with poor quality and lack of utilization of surgical care.^1^^,^^2^ Though the volume of surgery is growing worldwide, the safety and timeliness of care have not been optimized. Many low- and middle-income countries lag in meeting the global target for emergency and essential surgical care (i.e., 5,000 surgeries per 100,000 population per annum).^3^ Improving performance efficiency within the surgical care delivery system is one of the major potential ways to increase timely access to surgical care and eventually reduce the high surgical mortality and morbidity.^4^^,^^5^ The analysis by Jordi et al. of progress in countries meeting universal health coverage goals emphasized that an efficient health system is key for using resources, however insufficient, to positively impact health outcomes.^6^

Additionally, given that efficiency of care is a health care quality dimension, health facilities might benefit from closely monitoring system efficiency indicators. Perioperative (pre-surgery, intra-surgery, and post-surgery) system performance measures include surgical volume, operating room (OR) output, cancellation of elective surgeries, in-hospital wait time, first case on time, and patient turnover time (TOT), among other performance indicators (Table 1).^6^^,^^7^

**TABLE 1.** Key Surgical System Efficiency Indicators Adapted From the Saving Lives Through Safe Surgery Program in Ethiopia, 2016–2020

Indicators	Description	Classification or Benchmark
**OR productivity** ^a^	Definition: Total count of major, elective, and emergency surgeries^b^ performed in a health facility in target OR rooms/tables during the study period (i.e., 10 working days), excluding surgeries booked but canceled. Working schedule for elective surgeries is Monday–Friday, but the start times may vary. For this study, we used 6:00 am as the time when the night shift ends. Official work start time for civil servants is 8:30 am.	Optimum: ≥2 surgeries per day in a major OR
**Functional operating table**	Definition: A surgical table actively used by the surgical team in an OR during the study period (i.e., 10 working days). Nonfunctioning OR/table is an operating room that has been closed or a room/table that has not been used for a major surgery in the previous 10 days each day during the study period.	Functional: available for surgery 24 hours a day, 7 days a week
**SIST**	Definition: A time record for surgical incision of an elective surgical procedure scheduled for the first patient in a typical operation day (also known as a pacemaker case). In this study, the OR day starts at 6:00 am. A retrospective audit of SIST (HH:MM) was conducted by taking 10 randomly selected charts of the first-case surgery performed in the previous 90-day study period. Predefined SIST is a facility-defined specific time for first-case SIST, in writing, or communicated to surgical teams on the same.	Early, earlier than 9:00 am. Late, 9:00 am or later
**Turnover Time**	The time lapse between 2 consecutive elective surgical procedures (i.e., the time difference between the first surgical case end time and the immediate next case). Incision start time was recorded on the surgical logbook or anesthesia sheet. In this study, a retrospective chart review of 10 patient pairs (a total of up to 20 charts from each health facility) of consecutive post-surgical patients was conducted.	Acceptable: ≤30 minutes Prolonged: >30 minutes^c^ Needs immediate improvement: >60 mins
**Cancellation of elective surgery**	A nonemergent surgical procedure that is booked for operation but did not enter the theater or could not be operated on that day for various reasons. Cancellation rate was calculated as the number of elective surgeries booked for a given operation day and not operated on (numerator) divided by the total number of elective surgeries scheduled for the same day (denominator).	Acceptable rate: <5% Needs improvement: 5%–10% Needs immediate improvement: >10%
**In-hospital surgery waiting time**	The total duration of time lapsed from the time of admission to surgical wards until the recommended elective surgery, in the same facility. Not applicable for emergency surgery. A retrospective audit of time lapsed for individual surgical patients was conducted. Data aggregated for analysis and reporting purposes.	Timely, <24 hours Delayed, ≥24 hours

Abbreviations: OR, operating room; SIST, surgical incision start time.

^a^ In this study, OR productivity is synonymous with OR output.

^b^ Major surgery is any intervention occurring in a hospital OR involving the incision, excision, manipulation, or suturing of tissue, usually requiring regional or general anesthesia or sedation in a health care setting; this excludes all minor surgeries. Elective surgery is a nonemergent surgical procedure that is indicated for medical, surgical, or reconstructive purposes and could be delayed for at least 24 hours.

^c^ Turnover time longer than 30 minutes may indicate loss of surgical system efficiency and may indicate wastage of resources.

The Ministry of Health of Ethiopia’s national surgical care strategy (2016–2020) aimed to improve the quality and operational standards for the delivery of surgical care and defined surgical system performance metrics. However, no nationwide evaluation has been conducted to study the surgical system performance in public and private health facilities across Ethiopia. The few facility-based quality improvement reviews that have been conducted missed several efficiency metrics and suffered from national representation or generalization. Thus, the Ministry of Health and collaborating partners evaluated surgical system efficiency to document the overall surgical system performance and pinpoint the major factors influencing operative productivity in public and private health facilities. The evidence may inform future efforts to improve access to efficient surgical care. Lastly, quality improvement often fails due to poor implementation. A systematic review of quality improvement interventions by White et al. emphasized the need to strengthen the implementation strategies to further enhance the safety and quality of perioperative care.^8^^,^^9^

Surgical system efficiency was evaluated to document the system performance and pinpoint factors that influence operative productivity.

## METHODS

To expand access to timely and safe surgical care, Ethiopia implemented a 5-year surgical care strategy (2016–2020) and the Saving Lives Through Safe Surgery program. Key surgical system performance indicators were tracked from the national DHIS2 in public health facilities across the country.

### Study Design and Targets

A facility-based cross-sectional study design with retrospective record review was conducted to evaluate the OR performance and overall surgical system efficiency in 163 public and private health facilities in Ethiopia from December 2020 to June 2021.

### Sampling Procedure and Sample Size

A multistage stratified random sampling technique was used to recruit public and private health facilities. The required minimum sample size was calculated based on a single population proportion formula, (n=z^2^pq/e^2^/1+(z^2^pq/e^2^*N), for a finite population with a 50% population proportions, 5% margin of error, and 95% level of confidence. Of the total of 282 public health facilities that provided surgical care and routinely reported to the national DHIS2, 26 were specialized hospitals, 75 were general hospitals, and 181 were public primary health care units (PHCUs), of which there were 172 primary hospitals and 9 health center OR blocks. From a pool of health facilities that provided surgical care and routinely reported to the DHIS2, 163 public health facilities were sampled. The sample size for each stratum of PHCUs was calculated using the proportion-to-size allocation method and distributed as 15 specialized hospitals, 43 general hospitals, and 105 primary hospitals.

All sample health facilities that provided surgical care, reported to the national DHIS2, and presented records of the surgeries performed for the 90 days before data extraction were included. All minor surgeries and health facilities that did not provide elective (nonemergent) surgical care were excluded.

Because private facilities with surgical services did not implement the national Saving Lives Through Safe Surgery program and were not reporting surgical care performance data in the DHSI2 report, 45 private health facilities providing surgical care were also enlisted. Of those, 40 private health facilities were purposively selected, making a total of 203 (163 public and 40 private) sample study facilities recruited (Figure).

**FIGURE:**
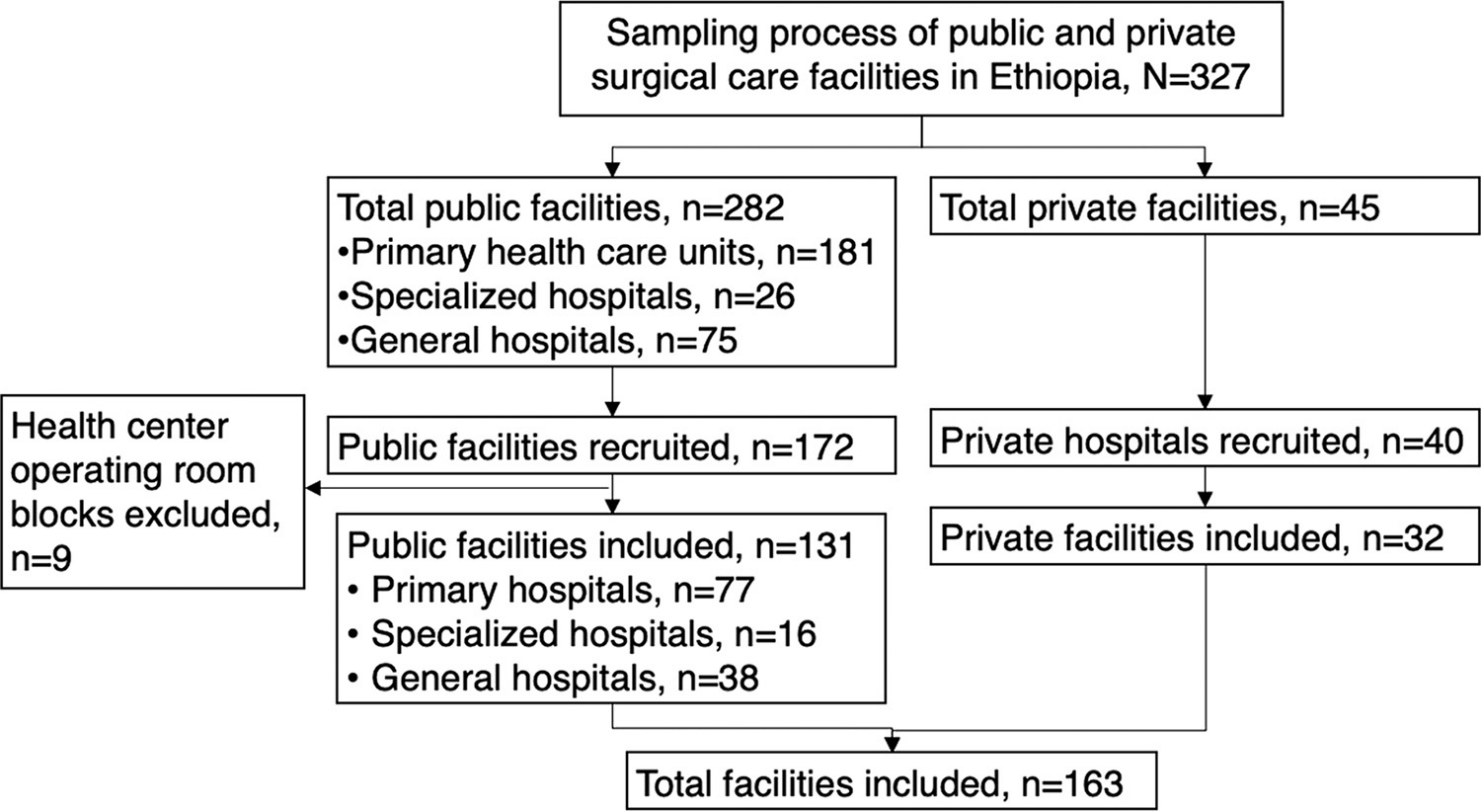
Sampling Process Applied in Evaluation of Surgical System Efficiency and Operative Productivity in Sample Public and Private Health Facilities in Ethiopia, December 2020 to June 2021

Initially, the study included 172 health facilities to evaluate (which accounted for 84.7% of the estimated initial sample size of 203), but 9 health centers with OR blocks were excluded from efficiency analysis due to lack of elective surgery service and data, leaving a total of 163 total facilities evaluated (131 public and 32 private health facilities). Each hospital from each stratum was selected by a simple lottery method. However, due to internal conflict and limited transport accessibility in some parts of Ethiopia, it was not feasible to access all randomly selected health facilities. Consequently, some health facilities were replaced with suitable alternatives under the assumption of homogeneity within the same stratum. Because of travel restrictions in conflict zones in Tigray region, only a few study facilities were included in the aggregate analysis.

### Data Variables

The key dependent efficiency variables are OR productivity or surgical volume. Independent variables include functionality of the OR, surgical incision start time (SIST), TOT, cancellation rate, and in-hospital surgery wait time (Table 1).

### Data Collection Tools and Procedure

The data collection tool, adapted from the World Health Organization’s Surgical Assessment Tool, was pretested and used to capture the required data.^10^ The routine DHIS2 surgical care service database was used to abstract surgical data for public health facilities. In both public and private health facilities, surgical registers, patient charts, and audit reports were reviewed. Key efficiency data (surgical volume, OR output, first-case SIST, TOT, cancellation rate, and in-hospital wait time) were collected for the 90 days before record review. The OR output data were collected for the 10 days before data collection.

Thirteen experienced surgical clinicians were trained to abstract efficiency data from the DHIS2 database, surgical service registers, patient charts, and quality audit reports. During data collection, precautionary measures recommended to prevent COVID-19 transmission, such as wearing a face mask, using hand sanitizers, and maintaining physical distance, were observed.

### Data Analysis

Data were checked on a weekly basis for completeness, correctness, and consistency and archived. Data were cleaned and entered into a Redcap study database. Data were exported to Stata 15 software for computing descriptive statistics (proportion, mean with standard deviation, and median with interquartile range) and compared to categorical variables by running a chi-square test. Statistical significance was declared at *P*<.05. A bivariable analysis was conducted, and the independent variables that demonstrated association with the dependent variable, OR productivity, were inputted into a multivariable regression analysis model. The type of health facility, level of care (primary and nonprimary health facility), and incision time were omitted from the multivariable analysis for collinearity reasons.

### Ethical Approval

Ethical clearance was secured from the Armauer Hansen Research Institute Institutional Review Board (AHRI PO/35/20]. The Ministry of Health of Ethiopia issued a letter of support to study regions and health facilities.

## RESULTS

### Health Facility Characteristics

In total, the study evaluated 163 sampled health facilities for the efficiency of surgical systems, comprising 80.30% (n=131) public and 19.63% (n=32) private health facilities. When disaggregated by level of care, public primary hospitals (47.23%, n=77) accounted for the majority of the study facilities.

### Operative Productivity

The surgery OR output per OR table was estimated based on the surgery volume for 10 days and showed that an average of 2 major surgeries were performed per table per day. Public specialized hospitals and private facilities contributed to 45.06% (n=4,104) and 18.27% (n=1,664), respectively (Table 2). The independent variables that showed association in a bivariable analysis were input to a multivariable regression analysis model; however, none of the predictor/independent variables showed significance.

**TABLE 2.** Surgical Operative Productivity in Public and Private Health Facilities in Ethiopia, December 2020 to June 2021

Level of Health Facilities	Facilities, No.	Average OR Tables per Facility, No.	Total Surgeries in 10 Days, No. (%)	OR Output (Productivity)^a^	
				Average Surgeries per OR Table for 10 Days, No.	Average Surgeries per OR Table per Day, No.
Public specialized hospital	16	8.34	4,104 (45.06)	25	3
Public general hospital	38	2.97	2,031 (22.23)	18	2
Public primary hospital	77	1.7	1,309 (14.37)	10	1
Private hospital	32	3.25	1,664 (18.27)	16	2
Total	163	3	9,108 (100)	18.6	2

Abbreviation: OR, operating room.

^a^ Note that the complexity of the surgical procedure and comorbidities patients had were not factored during analysis of operative performance.

### Functional Operating Tables

In the study facilities, 84.11% (482 of 573) of the operating tables were functional, and a total of 68,596 major surgeries were performed. Private hospitals performed 17.62% of these surgeries. Public specialized hospitals ran the highest number of functioning ORs with an average size of 8 tables per each public specialized hospital (Table 3).

**TABLE 3.** Characteristics of Select Surgical Inputs in Public and Private Health Facilities in Ethiopia, December 2020 to June 2021^a^

Level of Care, Type of Facility	Facilities, No. (%)	OR Tables, No. (%)	Functioning OR Table, No. (%)	Average OR Table per Facility	Surgical Beds, No. (%)	**Major Surgery,** **No. (%)**
Public specialized hospitals	16 (9.80)	158 (27.57)	134 (27.80)	8.34	1,375 (35.13)	22,420 (32.68)
Public general hospitals	38 (23.31)	125 (21.81)	113 (23.44)	2.97	1,139 (29.01)	17,665 (25.75)
Public primary hospitals	77 (47.23)	174 (30.36)	131 (27.17)	1.7	726 (18.55)	16,425 (23.94)
Private hospitals	32 (19.63)	116 (20.24)	104 (21.57)	3.25	674 (17.22)	12,086 (17.62)
Total	163 (100)	573 (100)	482 (100)	3	3,914 (100)	68,596 (100)

Abbreviations OR, operating room.

^a^ Of the primary health care units that were evaluated, 9 health center OR blocks were excluded from efficiency analysis due to lack of elective surgery service and the data.

Public specialized hospitals ran the highest number of functioning ORs with an average size of 8 tables per each public specialized hospital.

### Surgical Incision Start Time of the First Surgery

Only 31.29% (51 of 163) of the study facilities posted a SIST for the first elective surgery in a working day. However, of the total 881 surgery incision times audited, 19.86% (n=175) of the first-of-the-day elective surgeries were started late after 10:01 am, while 33.82% (n=298) of the surgeries had their incision time between 6:00 am and 8:30 am. The proportion of facilities with a predefined operation start time was high in public generalized and specialized hospitals, 42.11% and 56.25%, respectively (Table 4).

**TABLE 4.** Surgical Incision Start Time for the First Patients of an Operation Day in Public and Private Health Facilities in Ethiopia, December 2020 to June 2021

Level of Care, Type of Facility	Facilities, No.	Predefined SIST, No. (%)	Total Charts Audited, No. (%)	SIST				
				6:00 am–8:30 am, No. (%)	8:31– 9:00, No. (%)	9:01– 9:30, No. (%)	9:31– 10:00, No. (%)	After 10:01 am, No. (%)
Public specialized hospitals	16	9 (56.25)	135 (15.32)	63 (46.67)	27 (20.00)	26 (19.26)	12 (8.90)	7 (5.20)
Public general hospitals	38	16 (42.11)	300 (34.05)	122 (40.66)	60 (20.00)	51 (17.00)	33 (11.00)	34 (11.33)
Public primary hospitals	77	18 (23.37)	276 (31.33)	58 (21.01)	19 (6.89)	62 (22.46)	59 (21.37)	78 (28.26)
Private hospitals	32	8 (25.00)	170 (19.30)	55 (32.35)	21 (12.35)	23 (13.52)	15 (8.82)	56 (32.94)
Total	163	51 (31.29)	881 (100)	298 (33.82)	127 (14.41)	162 (18.39)	119 (13.5)	175 (19.86)

Abbreviation: SIST, surgical incision start time.

### Turnover Time

The aggregate average TOT computed from 305 consecutive patient-pairs (a total of 610 surgeries) was 50.25 minutes, and 48.52% (n=296) of the turnovers were completed within 30 minutes, while 11.47% (n=70) took longer than 90 minutes. TOT varied by type of facility; public hospitals had shorter TOT, 40.24–40.5 minutes, than private hospitals, 79.52 minutes (Table 5).

**TABLE 5.** Turnover Time Between Consecutive Elective Surgeries in Public and Private Health Facilities in Ethiopia, December 2020 to June 2021

Level of Care, Type of Facility	Facilities, No.	Total Charts Audited, No.	Turnover Time, Minutes				
			**<**30, No. (%)	31–60, No. (%)	61–90, No. (%)	**>**90, No. (%)	Average Turnover Time
Public specialized hospitals	16	104	54 (51.90)	32 (30.76)	14 (13.46)	4 (3.84)	40.24
Public general hospitals	38	214	106 (49.53)	76 (35.51)	20 (9.34)	12 (5.60)	40.76
Public primary hospitals	77	158	68 (43.03)	44 (27.84)	26 (16.45)	20 (12.65)	40.5
Private hospitals	32	134	68 (50.76)	30 (22.38)	2 (1.49)	34 (25.37)	79.52
Total	163	610	296 (48.52)	182 (29.83)	62 (10.16)	70 (11.47)	50.25

### In-Hospital Surgery Wait Time

Only 45.93% of the study health facilities routinely reported on in-hospital surgery wait time, and the mean wait time was 45.40 hours. The mean wait time varied by the level of health care; the longest mean wait time was observed in public generalized hospitals, 68.36 hours, while the shortest mean wait time was recorded in private hospitals, 18.33 hours (Table 6).

**TABLE 6.** In-Hospital Waiting Time for Elective Surgeries in Public and Private Health Facilities in Ethiopia, December 2020 to June 2021

Level of Health Facility	Facilities, No.	Facility Reports on Wait Time, No. (%)	In-Hospital Surgery Wait Time, Hours			
			**<**24, No. (%)	24–72, No. (%)	**>**72, No. (%)	Average±SD
Public specialized hospitals	16	10 (62.50)	4 (40.00)	4 (40.00)	2 (20.00)	59.90±27.85
Public general hospitals	38	23 (60.52)	10 (43.48)	10 (43.48)	3 (13.04)	68.36±26.80
Public primary hospitals	77	31 (40.26)	12 (38.71)	16 (51.61)	3 (9.68)	36.80±46.60
Private hospitals	32	15 (46.87)	7 (46.67)	8 (53.33)	0 (0)	18.33±3.06
Total	163	79 (45.93)	21 (43.75)	22 (45.83)	5 (10.42)	45.40±9.25

Abbreviation: SD, standard deviation.

### Cancellation Rate

The aggregate elective surgery cancellation rate abstracted from facility self-report archived in the national DHIS2 database was 5.2% (ranging from 2.8% to 7.7%). The cancellation rate disaggregated by level of care ranges from 2.5% (ranging from 0.5% to 5.6%) in private hospitals to 14.6% (ranging from 6.1% to 22.9%) in public specialized hospitals.

### Analysis of Factors and Their Associations With Operating Room Productivity

#### Surgical Incision Start Time

The presence of a predefined SIST was associated with an optimum OR productivity (*P*=.004) (Table 7). It was also shown that first-case incision started before 9:00 am had a significant association with shorter surgery wait times (*P*=.016) (Table 8).

**TABLE 7.** Association Between Operating Room Productivity and Presence/Absence of Predefined Surgical Incision Start Time for the First Patient in Public and Private Health Facilities in Ethiopia, December 2020 to June 2021

	Operative Productivity			Association, Significance
	Optimum,^a^ No. (%)	Suboptimum, No. (%)	Total, No.	
Utilized predefined surgical incision start time				
Yes	20 (54.05)	5 (18.52)	25	*P*=.004
No, absent	17 (45.94)	22 (81.48)	39	
Total	37 (100)	27 (100)	64	

^a^ Optimum operative productivity is an average of 2 or more surgeries performed per day in a major operating room.

**TABLE 8.** Association Between In-Hospital Surgery Wait Time and Surgical Incision Start Time in Public and Private Health Facilities in Ethiopia, December 2020 to June 2021

	In-Hospital Surgery Wait Time, Hours			Association, Significance
	Timely **<**24, No. (%)	Delayed **≥**24, No. (%)	Total, No.	
Surgical incision start time				
Early: Before 9:00 am	16 (94.12)	14 (60.87)	30	*P*=.016
Late: 9:00 am or after	1 (5.88)	9 (39.13)	10	
Total	17 (100)	23 (100)	40	

#### In-Hospital Surgery Wait Time

Short in-hospital surgery wait time was not associated with optimum operative productivity (*P*=.673) (Table 9).

**TABLE 9.** Association Between Operative Productivity and In-Hospital Surgery Wait Time in Public and Private Health Facilities in Ethiopia, December 2020 to June 2021

	In-Hospital Surgery Wait Time, Hours			Association, Significance
	Timely **<**24, No. (%)	Delayed **≥**24, No. (%)	Total, No.	
Operative productivity				
Optimum^a^	9 (64.29)	12 (57.14)	21	*P*=.673
Suboptimum	5 (35.71)	9 (42.86)	14	
Total	14 (100)	21 (100)	35	

^a^ Optimum operative productivity is an average of 2 or more surgeries performed per day in a major operating room.

#### Turnover Time

No association was demonstrated between operative performance and the TOT between successive surgeries (*P*=.981) (Table 10).

**TABLE 10.** Association Between Operative Productivity and Turnover Time Between Successive Surgeries in Public and Private Health Facilities in Ethiopia, December 2020 to June 2021

	Operative Productivity			Association, Significance
	Optimum,^a^ No. (%)	Suboptimum, No. (%)	Total	
Turnover time				
Acceptable: ≤30 minutes	7 (38.89)	5 (38.46)	12	*P*=.981
Prolonged: >30 minutes	11 (61.11)	8 (61.54)	19	
Total	18 (100)	13 (100)	31	

^a^ Optimum operative productivity is an average of 2 or more surgeries performed per day in a major operating room.

## DISCUSSION

OR output is a strong predictor of surgical system efficiency and an indirect measure of timeliness of access to surgical care.^11^ In this study, despite the functionality of the ORs (84.11%), the surgical system efficiency and OR output in both public and private health facilities in Ethiopia is low, with an aggregate average of 2 surgeries per day per OR table. Low operative output can result from a loss of efficiency due to distractions, interruptions, and disruptions during emergency surgeries and a surgical climate that may impact operative efficiency.^12^ In their prospective review of operative efficiency of 299 surgeries conducted in Australian hospitals, Wallace et al. indicated that a significant proportion of OR time was spent on nonoperative tasks, and time spent for surgical preparation represented 42.4% (37.28 minutes), operating time 40.1% (35.28 minutes), and finishing-up time 17.5% (15.43 minutes).^13^

Despite the functionality of the ORs, the surgical system efficiency and OR output in both public and private health facilities is low.

Compared to public specialized and general hospitals, public primary and private health facilities performed the lowest number of surgeries. Additionally, the study documented a longer patient TOT, 79.52 minutes, in private health facilities. Some of the explanations for the high performance of public specialized tertiary-level care facilities could be the availability of a relatively higher number and mix of surgical professionals, the availability of intensive care unit setup, established hospital and surgical management system and leadership practices, and a lower cost of surgical care, among other factors.

In contrast, private health facilities scored favorably for demonstrating the lowest cancellation rate and in-hospital wait time for surgery. Although this study did not investigate other factors that influence the OR productivity in private health settings, the possible reasons may include but are not limited to criteria for admission of surgical cases, type of surgery performed, availability of a multidisciplinary surgical team, fear of legal litigation, and access to blood and blood products.

Here, one could argue that potentials in primary and private facilities could be met by addressing the bottlenecks of productivity. This agrees with a technical efficiency analysis conducted in Ethiopia by Mann et al. that evaluated the minimum level of resources for a unit of service output, in this case, surgical volume, and presented that only 29% of the 24 primary hospitals were technically efficient. The average pure technical efficiency score among the inefficient primary hospitals was 55%, implying that, on average, the inefficient primary hospitals could reduce their input by 45% without reducing output.^14^

OR productivity is influenced by an interplay of multiple independent factors that may be interrelated. Select independent variables demonstrated a strong association in a bivariable analysis but lacked significance in a multivariable regression analysis. This could be due to several reasons, including small sample size, the nature of independent variables, or other factors that may impact the multivariable association. This may call for robust research that includes a larger sample size and controls confounding factors.

### Surgical Incision Start Time Was Associated With Operative Productivity

This study demonstrated a strong association between SIST and OR productivity (*P*=.004). It is alarming to note that most health facilities lacked a predefined incision start time and that nearly one-fifth of the surgeries were delayed (i.e., first-case incision performed after 10:00 am). Delay in operation start time is not uncommon in Ethiopian surgical settings. For example, Samuel et al. prospectively studied 933 elective surgeries performed in a tertiary hospital in Ethiopia and found that the elective surgery start time was delayed in 93.40% of cases with a mean start time of 9:41±60 minutes.^15^ Another study of the performance of 9 ORs in a tertiary teaching hospital in Addis Ababa by Tiruneh et al. reported an average delay of 43 minutes from the agreed 8:00 am, and only 2.50% of the operations were started as per the schedule communicated before the surgery.^16^

The literature described multiple interrelated factors to explain delays in surgery start time and overall OR productivity. Wright et al. reported surgeon and anesthesiologist unavailability and lack of preparedness of patients as the most common reasons for not starting on time.^17^^,^^18^ Team briefings and motivation may also play a role in the overall productivity of the OR. Martin et al. published evaluation results of a pre-OR safety briefing and a modest performance pay incentive for on-time starts (>90% compliance) for surgeons at a Veterans Affairs Medical Center. Before implementing either the pre-OR briefing or pay incentive, only 15% of cases started on time, and after implementation, 72% of cases started on time (*P*<.001). This initiative not only significantly improved OR utilization but also reduced unnecessary costs.^19^ Of note, the study’s tertiary-level teaching hospitals performed a higher number of elective surgeries during off-peak shift hours, between 6:00 am and 8:30 am. This could be because tertiary teaching hospitals have more surgical staff during off-work hours and may receive more case referrals and emergencies from other health facilities.

### Turnover Time and Cancellation Rate Were Not Associated With Operating Room Productivity

Compared to the national recommended standard of 30 minutes, the mean aggregate TOT computed in this study of 50.25 minutes was much longer. A TOT of more than 30 minutes may indicate a loss of surgical process efficiency or a waste of resources. However, the TOT was not associated with OR productivity in this study setting. The possible reasons for loss of efficiency from an extended TOT could arise from a lack of standardization of surgical processes, ineffective communication across a multidisciplinary surgical team, weak accountability, rotation of the surgical team, and inadequate cleaning supplies. According to a retrospective review by Kwadwo et al. of 2,714 surgeries, consecutive surgeries performed by the same surgical team produced a significantly lower TOT (*P*<.0001), and it is highly recommended to perform consecutive elective surgeries by the same OR team.^20^ Further study on local factors influencing TOT and feasible strategies for optimizing transition time between operational processes could be rewarding.^21^^,^^22^

The aggregate cancellation rate of 5.20% obtained in this study was lower than the rate reported by other studies conducted in Ethiopia and other resource-limited settings. This could be because the initial part of the study period overlapped with the onset of the COVID-19 pandemic, and the number of elective surgeries scheduled, and overall surgical volume documented in the early phases of the pandemic was on the lowest side compared to the pre-pandemic period. A systematic review and meta-analysis of surgery cancellation in Ethiopia by Birhanu et al. estimated a pooled cancellation prevalence of 21.41% (95% confidence interval=12.75, 30.06) based on studies that involved 5,591 surgeries.^23^ Likewise, Feleke et al. documented a 25.6% cancellation rate among the 326 surgical patient schedules for elective surgery,^24^ and other tertiary-level care teaching hospitals in Ethiopia showed even higher cancellation rates of 33.90% and 35.80%.^25^

High cancellation rates remain a concern for surgical settings in both low-income and high-income countries. In Burkina Faso, Lankoande et al. proactively evaluated the incidence of elective surgery cancellation in a teaching hospital and showed that 36.90% (38 of 103) of surgeries were delayed, of which 9.70% (n=10) were canceled and another 27.20% (n=28) were delayed. Hospital-related cancellation accounted for 63.90%. The authors also argued that cancellations were avoidable in 68.5% of cases.^26^

In our study, the more common reported reasons for cancellation were grouped under medical reasons (e.g., patient unfit and reported acute illness) and shortage of blood and blood products, 35% and 25%, respectively. Additional reasons for cancellation were delayed COVID-19 test turnaround time, 4.48% (n=7); interrupted water supply, 3.21% (n=5); interrupted oxygen supply, 1.90% (n=3); and interrupted electric power supply, 1.28% (n=2).

A study in Tikur Anbessa Specialized Hospital in Ethiopia, where 35.8% of elective surgeries were canceled, reported that the most common reasons for cancellation were inadequate OR time and lack of patient preparation, 8.70% and 7.70%, respectively.^15^ Feleke et al. argued that surgical cancellations were primarily due to patient-related and administrative factors, 31.32% and 26.5%, respectively.^24^ Dhafar et al. retrospectively audited a total of 120 ORs in 25 hospitals in Saudi Arabia and reported that among the 7.60% (1,238 of 16,211) surgeries canceled, the major contributing factors were related to the patient (42.81%), facility (20.03%), and incomplete work-up (9.45%).^27^ In these studies, the medical and patient-related reasons accounted for 13.90% and 13.34%, respectively.^23^^,^^28^^,^^29^

Sadly, according to an integrative surgical literature review by Narmeen et al., most cancellations were avoidable.^30^

### In-Hospital Surgery Wait Time Was Significantly Associated With First-Case Incision Time

This study demonstrated that the duration of in-hospital wait time is significantly associated with the first-case incision start time (*P*=.016). However, though other studies have indicated that in-hospital wait time is a strong predictor of surgical efficiency, there was no strong association between the in-hospital wait time and OR productivity (*P*=.673). Of those facilities that reported wait times, the observed mean wait time, 45.4±9.25 hours, was much longer than the national limit of 24 hours. The unnecessarily long in-hospital delay in public specialized hospitals may be partially explained by the high rate of surgery cancellations in those hospitals (14.6%) and the burden that tertiary-level care hospitals shoulder in terms of high bed occupancy by sizable proportion of surgical admissions and case referrals. Unfortunately, in-hospital emergency and elective surgical delays are not uncommon in resource-limited settings. In Malawi, Maine et al. showed that 281 of 764 (36.80%) emergency surgeries were delayed.^31^ Although the factors contributing to delays could be many and interrelated, a prospective observational study by Ray and Kirtania on wait time in India presented that the client’s age, physical fitness, and economic stature were factors significantly influencing wait time (*P*<.05).^32^

This study demonstrated that the duration of in-hospital wait time is significantly associated with the first-case incision start time.

### Impact of Inefficient Surgical Systems on Quality of Care and Patient Safety

Inefficient surgical systems not only affect the targets set for addressing the huge unmet need for essential and emergency surgical care in low- and middle-income countries but also jeopardize patient safety.^7^^,^^33^^,^^34^ For example, Maine et al. reviewed 764 emergency surgeries in Malawi to show that a delay of surgery of more than 24 hours was associated with increased risk of surgical complications, including generalized peritonitis (relative risk=4.49; 95% confidence interval=1.69, 11.95; *P*=.005) and gastrointestinal perforation (relative risk=3.73, 95% confidence interval=1.25, 11.08; *P*=.018), and an increased risk of mortality.^31^

This warrants a renewed commitment to strengthening the safety and quality of perioperative care and introducing effective implementation strategies. At times, lack of agreement on efficiency indicators makes comparison of research findings on system efficiency very challenging, and further research on performance efficiency metrics and determinant factors is highly encouraged.^35^

### Strengths and Limitations

This was the first nationwide evaluation of surgical system efficiency and OR productivity in public and private surgical facilities at all levels of health care in Ethiopia.

Because this evaluation may not include every system efficiency metric for pre-surgery, intra-surgery, and post-surgery processes and lacks a qualitative study component, deeper insights into the factors influencing surgical system efficiency may not be captured well. This may make generalizability of some of the findings less applicable in other surgical care settings. Though significant efforts were made to minimize facility selection bias, the omission of sample facilities in conflict and war zones and subsequent replacement by other similar facilities, as well as the interruption of surgical due to the COVID-19 pandemic, may have introduced some bias.

The initial phase of the study period overlapped with the onset of the COVID-19 pandemic, and the number of elective surgeries scheduled and performed has declined compared to the pre-COVID-19 period. However, in the later phases of the data collection period, health facilities introduced a surgical backlog clearance mechanism to maintain and increase surgical performance.

## CONCLUSIONS AND RECOMMENDATION

Though a majority of the ORs were functional, the surgical system efficiency in both public and private health facilities was low. OR productivity was affected by several factors, including SIST, patient TOT, cancellation rate, and in-hospital wait time.

OR productivity is strongly associated with having an agreement on the specific or defined time for starting an operation and in-hospital wait time. Further, first-case incision start time is strongly associated with in-hospital wait time for surgery. Therefore, facilities need to set the incision start time, continuously track operation start and end times and other efficiency metrics, and enhance compliance with national perioperative care guidelines.

The inefficiency of surgical systems because of a delay in first-case surgical operation, prolonged TOT, high cancellation rate, and long in-hospital wait time for surgery in both public and private health facilities calls for immediate action. If inefficiencies could be corrected, the larger investment in infrastructure, supplies, and even human resources could increase the return on investment made by the Government of Ethiopia. Inefficiencies and preventable delays need to be investigated further.

We recommend team-based efficiency optimization strategies and continuous monitoring of operating performance and patient safety across facilities.

## Notes

**Peer Reviewed**

**First published online:** February 9, 2024.
